# Overexpression of *OsGID1* Enhances the Resistance of Rice to the Brown Planthopper *Nilaparvata lugens*

**DOI:** 10.3390/ijms19092744

**Published:** 2018-09-13

**Authors:** Lin Chen, Tiantian Cao, Jin Zhang, Yonggen Lou

**Affiliations:** State Key Laboratory of Rice Biology & Ministry of Agriculture Key Lab of Agricultural Entomology, Institute of Insect Sciences, Zhejiang University, Hangzhou 310058, China; chenlin88@126.com (L.C.); caotiantiande@sina.cn (T.C.); zhangjin1369@tricaas.com (J.Z.)

**Keywords:** rice, *OsGID1*, gibberellin, herbivore-induced plant defenses, *Nilaparvata lugens*

## Abstract

Gibberellins (GAs) play pivotal roles in plant growth and development, and in defenses against pathogens. Thus far, how the GA-mediated signaling pathway regulates plant defenses against herbivores remains largely unknown. In this study, we cloned the rice GA receptor gene *OsGID1*, whose expression was induced by damage from the brown planthopper (BPH) *Niaparvata lugens*, mechanical wounding, and treatment with salicylic acid (SA), but not jasmonic acid. The overexpression of *OsGID1* (oe-GID1) decreased BPH-induced levels of SA, H_2_O_2_, and three SA-pathway-related WRKY transcripts, but enhanced BPH-induced levels of ethylene. Bioassays in the laboratory revealed that gravid BPH females preferred to feed and lay eggs on wild type (WT) plants than on oe-GID1 plants. Moreover, the hatching rate of BPH eggs on oe-GID1 plants was significantly lower than that on WT plants. In the field, population densities of BPH adults and nymphs were consistently and significantly lower on oe-*OsGID1* plants than on WT plants. The increased resistance in oe-GID1 plants was probably due to the increased lignin level mediated by the GA pathway, and to the decrease in the expression of the three WRKY genes. Our findings illustrated that the *OsGID1*-mediated GA pathway plays a positive role in mediating the resistance of rice to BPH.

## 1. Introduction

In natural ecosystems, the evolutionary arms race between plants and insects has been ongoing for more than 400 million years, and along the way plants have evolved diverse strategies to protect themselves [1,2,3]. When facing herbivore attack, plants identify the type of damage caused by herbivores and then trigger a signaling network comprising calcium signaling, mitogen-activated protein kinase (MAPK) cascades, and signaling pathways mediated by phytohormones, such as jasmonic acid (JA), salicylic acid (SA), and ethylene (ET) [4,5,6]. Once activated, this signaling network elicits plants’ production of defense compounds and enhances their resistance to herbivores. Recent research advances have highlighted how some growth-related phytohormones, such as auxins, brassinosteroids, cytokinins, and gibberellins (GAs), also regulate plant defenses [7].

GAs are a series of tetracyclic diterpenoid phytohormones, which play crucial roles in numerous processes underlying plant growth and development, including cell expansion and division, seed germination, and floral development [8]. By combining genetics, molecular biology, and plant physiology, key components acting in the regulation of GA biosynthesis, signal perception, and transduction have been identified [9], such as the GA receptor GID1 (GA-INSENSITIVE DWARF1) [10,11], DELLA proteins [12], and the F-box protein GID2 (GA-INSENSITIVE DWARF2) in rice [13]. Moreover, the GA–GID1–DELLA regulatory module, which controls GA signaling is well established [14]. In this module, GA signaling starts by binding bioactive GAs to the GA receptor GID1 to form a GA-GID1 complex [10,15]. Together with E3 ubiquitin-ligase SCF^SLY1/GID2^, the complex then stimulates the rapid degradation of DELLA proteins, the master repressors of the GA-signaling pathway, and activate the GA pathway [14,16]. As a key component of the gibberellin-signaling pathway, GA receptors have been cloned in many higher plant species and characterized using genetic approaches [17]. In rice, there is only one GA receptor gene, *OsGID1*, and it encodes a soluble hormone-sensitive lipase-like protein, whereas in *Arabidopsis*, there are three GA receptor genes, *AtGID1a*, *AtGID1b*, and *AtGID1c*, and these genes function redundantly [15,18].

In addition to its central role in regulating plant growth and development, GA-mediated signaling has also been reported to play a pivotal role in plant defenses against pathogens by directly or indirectly interacting with defense-related signaling pathways. In *Arabidopsis*, for instance, DELLAs were found to negatively mediate SA biosynthesis and signaling; such regulation enhances the plant’s resistance to necrotrophs and susceptibility to biotrophs [19]. In rice, the DELLA protein Slender Rice1 (OsSLR1) promotes defenses mediated by both SA and JA, and these enhance rice resistance to hemibiotrophic pathogens [20]. In addition, Tanaka et al. [21] reported that *gid1*, a gibberellin-insensitive dwarf mutant, showed increased resistance to blast fungus due to high levels of a probenazole-inducible protein (PBZ1). In contrast, less attention has been paid to the role of the GA-mediated signaling pathway in herbivore-induced plant defenses. Lan et al. [22] found that in *Arabidopsis*, DELLA proteins optimize and fine-tune plant defense and growth by suppressing plant defense hormones in the labial salivary secretions of caterpillars. In rice, the GA-mediated pathway has been found to positively regulate resistance to the brown planthopper (BPH) *Nilaparvata lugens* (Stål) [23]. Moreover, Zhang et al. [24] reported that silencing the DELLA gene *OsSLR1* enhances the resistance of rice to BPH in the laboratory and field by impairing JA and ethylene pathways, and by enhancing the levels of lignin. These new findings shed light on the role of GA-mediated signaling in plant defenses against herbivores. However, how the GA-mediated pathway regulates herbivore-induced defenses remains largely unknown.

Rice, one of the most important food crops in the world and eaten by more than half of the world’s population, suffers severely from many insect pests, including the phloem-sucking insect, brown planthopper (BPH) *Nilaparvata lugens* [25]. Previous studies of rice have revealed that the infestation of BPH gravid females changes the biosynthesis of many defense-related signals, such as JA, JA-Ile, SA, ET, and H_2_O_2_; in turn, these compounds modulate defense responses, for example, by influencing the production of herbivore-induced volatiles and the activity of TrypPIs [26,27,28]. Given the role of GA-mediated signaling in plant defenses, we cloned the rice GA receptor gene *OsGID1* (TIGR ID Os05g33730) and characterized its role in herbivore-induced defenses in rice. By combining molecular biology, reverse genetics, chemical analysis, and bioassays, we found that the overexpression of *OsGID1* decreases the BPH-induced accumulation of three WRKY transcription factor (TF) mRNAs, and of SA and H_2_O_2_, but increases ethylene levels. Moreover, the overexpression of *OsGID1* enhances the resistance of rice to BPH, suggesting the *OsGID1*-mediated GA signaling pathway also plays a role in resistance.

## 2. Results

### 2.1. Isolation and Expression Patterns of OsGID1

Based on the cDNA sequence of *OsGID1* in the MSU Rice Genome Annotation Project database, we cloned the *OsGID1* gene from a cDNA library of rice variety XS110 using PCR. The *OsGID1* gene contains an open reading frame of 1065 nucleotides, of which it consists of two exons and one intron (Supplemental Figure S1) and encodes a soluble GA receptor of 354 amino acids, which is mainly localized in the nucleus [10]. There are four single nucleotide polymorphisms (SNPs) in the coding sequence (CDS) of *OsGID1*, two of which are missense substitutions and the others are synonymous nucleotide substitutions (Supplemental Figure S1). To analyze the evolutionary history of OsGID1 and its orthologs from higher plant species, a phylogenetic tree was constructed using neighbor-joining analysis. According to the phylogenetic tree, OsGID1 shows the highest homology with GID1s from four monocotyledonous species: ZmGID1 in *Zea mays*, SiGID1 in *Setaria italic*, SbGID1 in *Sorghum bicolor*, and SoGID1 in *Saccharum officinarum*, sharing 80.79%, 84.46%, 82.91%, and 81.97% amino acid sequence identity with OsGID1 (Supplemental Figure S2).

**Figure 1:**
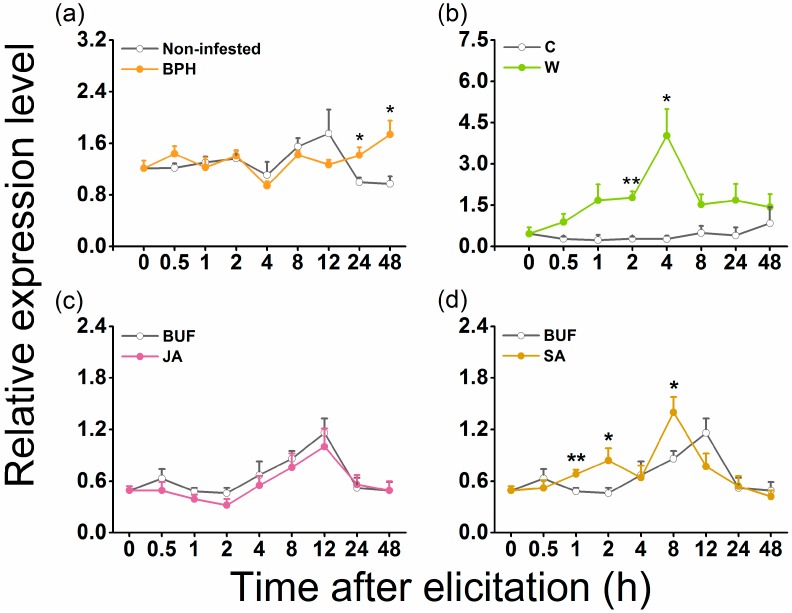
Quantitative real-time PCR (qRT-PCR) analysis of *OsGID1* transcript levels in rice after different treatments. Relative levels (means + SE, *n* = 5) of *OsGID1* transcripts in rice plants that were infested by gravid brown planthopper (BPH) females (**a**) or treated with mechanically wounding (**b**), jasmonic acid (JA) (**c**) or salicylic acid (SA) (**d**). Asterisks indicate significant differences between treatments and controls (* *p* < 0.05, ** *p* < 0.01, Student’s *t*-test).

Relative expression levels of *OsGID1* in response to different treatments were analyzed by quantitative real-time PCR (qRT-PCR). The expression of *OsGID1* was induced by gravid BPH females, at 24 and 48 h after infestation (Figure 1a); after mechanical wounding, expression levels increased rapidly and peaked at 4 h (Figure 1b). JA treatment had almost no influence on levels of *OsGID1* transcripts, whereas at some time points SA treatment significantly enhanced the expression of *OsGID1* (Figure 1c,d). These data indicate that *OsGID1* might mediate BPH-induced defenses in rice.

### 2.2. Overexpression of OsGID1

**Figure 2:**
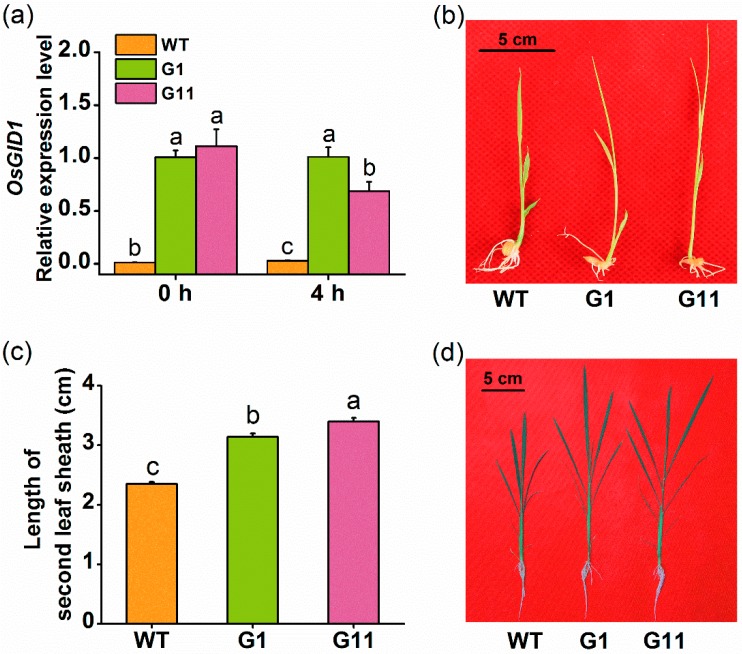
Relative expression levels of *OsGID1* in wild type (WT) plants, and in plants from lines expressing *OsGID1* and their growth phenotypes. (**a**) Relative levels (means + SE, *n* = 5) of *OsGID1* transcripts in oe-GID1 and WT plants 0 and 4 h after they were wounded (these two experiments (two time points) were performed separately); (**b**,**d**) Growth phenotypes of oe-GID1 and WT plants at day 10 and day 38 in the greenhouse, bar = 5 cm; (**c**) Mean length (+ SE, *n* = 30) of the second leaf sheath of 10-day-old oe-GID1 and WT plants. Different letters indicate significant differences between oe-GID1 lines and WT plants (Duncan’s multiple range test, *p* < 0.05).

To further investigate the function of *OsGID1* in herbivore-induced plant defenses, we generated rice plants with overexpressed *OsGID1* (oe-GID1), under the control of the cauliflower mosaic virus 35S promoter. Two T2 homozygous lines with a single insertion (Supplemental Figure S3), G1 and G11, were selected for further investigation. qRT-PCR analysis showed that levels of *OsGID1* transcripts in plants of the two oe-GID1 lines, G1 and G11, were 81.3–89.7 fold and 34.9–23.7 fold higher than those levels in wild type (WT) plants 0 and 4 h after mechanical wounding (Figure 2a). Similar to the results reported by Ueguchi-Tanaka et al. [10], transgenic plants from the lines expressing *OsGID1* exhibited a GA overdose phenotype: oe-*GID1* plants were taller and had longer leaves, compared to WT plants; moreover, in both 10- and 38-day-old seedlings of oe-GID1 plants, the second leaf sheath was significantly longer than the same sheath in WT plants (Figure 2b–d). At 38 days, the mass of the above-ground part of oe-GID1 lines, G1 and G11, increased by approximately 15% and 24%, respectively, compared to that of WT plants (Supplemental Figure S4).

### 2.3. Overexpressing OsGID1 Enhances the Resistance of Rice to BPH

**Figure 3:**
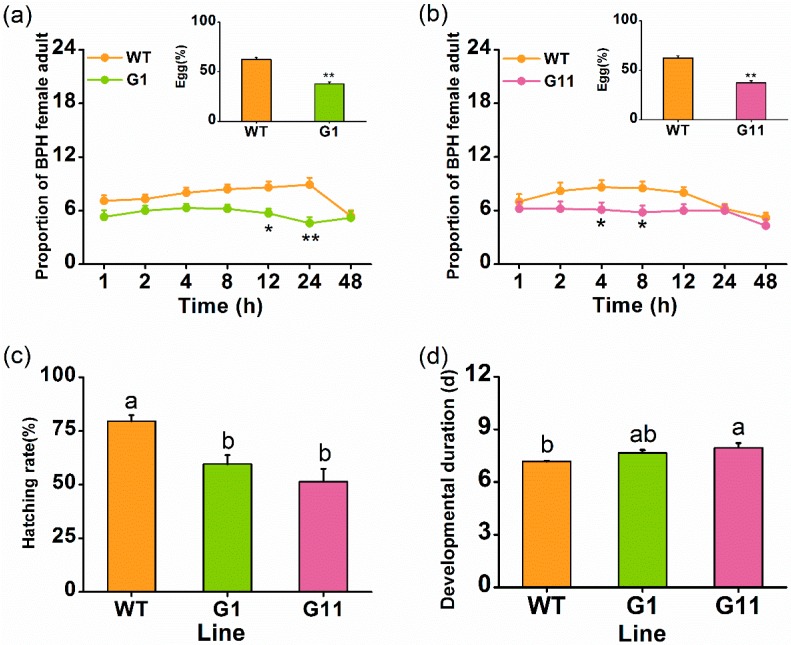
*OsGID1* positively mediates rice resistance to BPH. (**a**,**b**) Mean number of gravid BPH females per plant (+SE, *n* = 10) on pairs of plants (WT versus G1 or G11) in two-choice assays, 1–48 h after pairs were exposed to the herbivore. Inserts: Mean percentage of BPH eggs per plant (+SE, *n* = 10) on pairs of plants as stated above, 48 h after the release of BPH. (**c**) Mean hatching rate (+SE, *n* = 10) of BPH eggs on WT and oe-GID1 plants; (**d**) Mean developmental duration (+SE, *n* = 10) of BPH eggs on WT and oe-GID1 plants. Asterisks indicate significant differences in oe-GID1 plants, compared with WT plants (* *p* < 0.05, ** *p* < 0.01, chi-square test); different letters indicate significant differences between oe-GID1 lines and WT plants (Duncan’s multiple range test, * *p* < 0.05).

Previous studies have shown that GA signaling is involved in plant defense [7,24]; therefore, we examined whether the overexpression of *OsGID1* affects the resistance of rice to BPH. In a two-choice experiment, gravid BPH females preferred to feed and lay eggs on WT plants than on oe-GID1 plants (Figure 3a,b). Moreover, the hatching rate of BPH eggs on WT plants was dramatically higher than on oe-GID1 plants (Figure 3c). Finally, the developmental duration of BPH eggs on oe-GID1 plants showed longer than that on WT plants, although the difference between WT plants and G1 plants was not significant (Figure 3d). These results indicated that the overexpression of *OsGID1* enhanced the resistance of rice to BPH.

### 2.4. Overexpression of OsGID1 Suppressed the Expression of Three WRKY Transcription Factors

**Figure 4:**
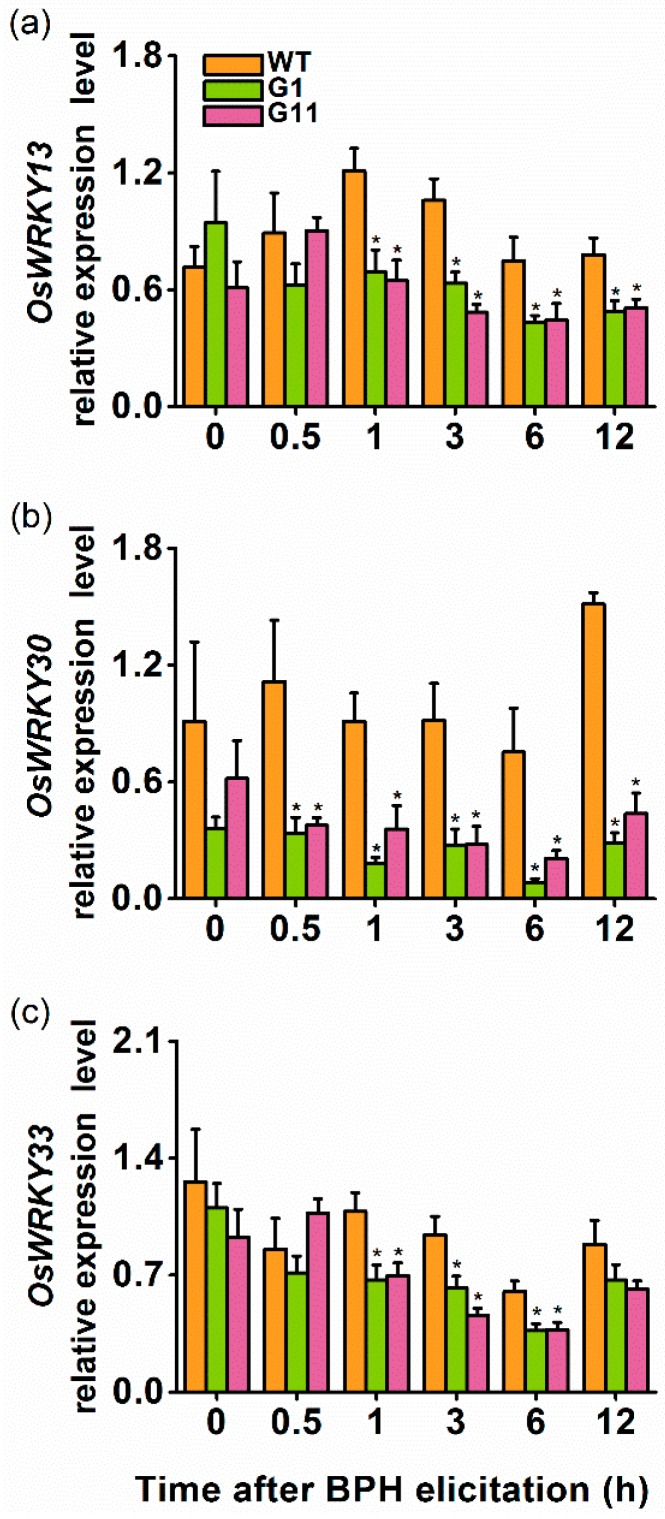
Overexpression of *OsGID1* decreases transcript levels of three WRKY TF genes. Relative levels (means + SE, *n* = 4–5) of *OsWRKY13* (**a**), *OsWRKY30* (**b**), and *OsWRKY33* (**c**) transcripts in oe-GID1 and WT plants after infestation by gravid BPH females. Asterisks indicate significant differences in oe-GID1 plants, compared with WT plants (Duncan’s multiple range test; * *p* < 0.05).

Three WRKY TFs—*OsWRKY13*, *OsWRKY30*, *OsWRKY33*—have been reported to be involved in the SA-mediated signaling pathway, and thus regulate rice defense [29,30,31]. Hence, we monitored mRNA levels of these TFs in plants of oe-GID1 lines, and in WT plants after infestation with gravid BPH females. The transcript levels of *OsWRKY13* did not differ between WT and oe-GID1 plants at 0 h and 0.5 h post BPH infestation. However, from 1 h to 12 h post BPH infestation, the transcript level of *OsWRKY13* was significantly lower in oe-GID1 plants than in WT plants (Figure 4a). Similarly, mRNA levels of *OsWRKY30* (Figure 4b) and *OsWRKY33* (Figure 4c) in oe-GID1 plants decreased from 0.5 to 12 h and from 1 to 6 h, respectively, post BPH infestation. These data suggest that *OsGID1* negatively regulated the transcript levels of the three WRKY genes.

### 2.5. Overexpression of OsGID1 Alters Accumulation of BPH-induced H_2_O_2_, SA and Ethylene

**Figure 5:**
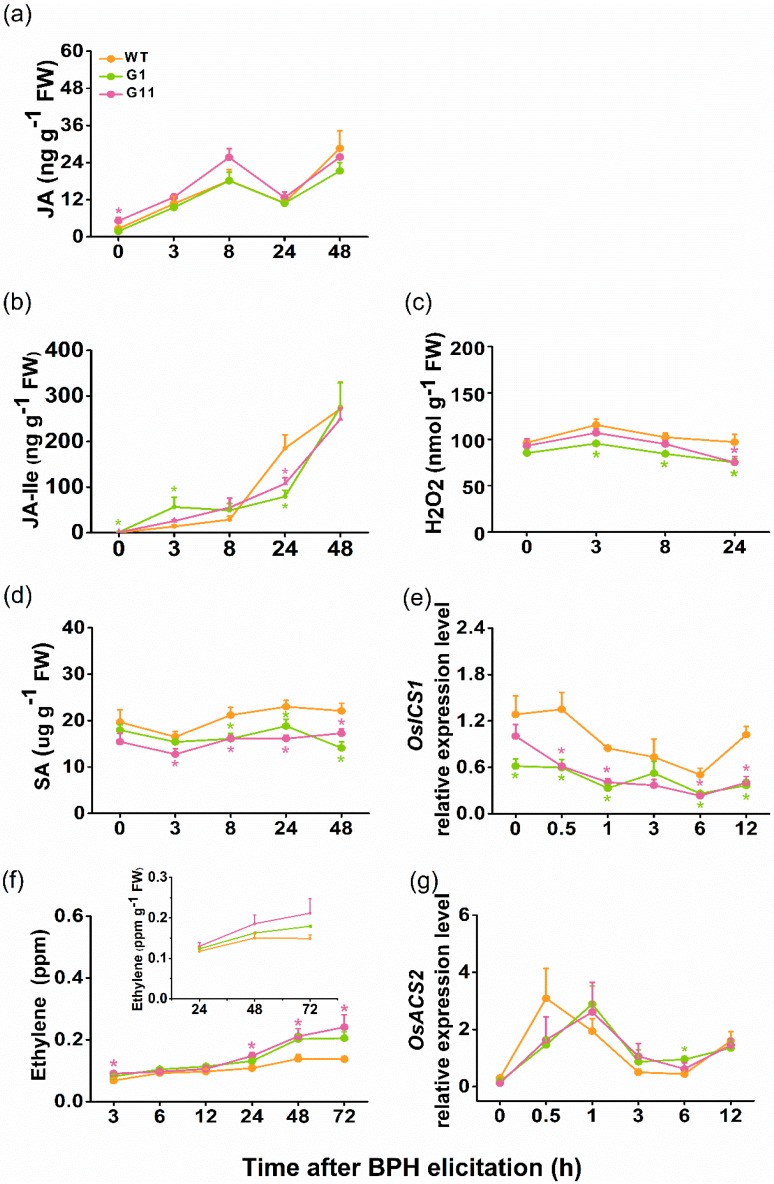
*OsGID1* regulates the accumulation of BPH-induced JA, JA-Ile, H_2_O_2_, SA, and ethylene. (**a**–**d**,**f**) Levels (means + SE, *n* = 4–8) of JA (**a**), JA-Ile (**b**), H_2_O_2_ (**c**), SA (**d**), and ethylene (**f**, insert: ethylene levels per g biomass) in/from oe-GID1 and WT plants at different times after they were infested by 15 gravid BPH females; (**e**,**g**) Relative levels (means + SE, *n* = 5) of *OsICS1* (**e**) and *OsACS2* (**g**) in oe-GID1 and WT plants at different times after they were infested by 15 gravid BPH females. Asterisks indicate significant differences in oe-GID1 plants, compared with WT plants (Duncan’s multiple range test; * *p* < 0.05; ** *p* < 0.01).

JA, JA-Ile, SA, H_2_O_2_, and ethylene have been reported to play important roles in plant defenses against herbivores [32,33,34]. Moreover, as reported previously, these signals have been implicated in defense responses of rice to BPH [35,36,37]. To test if JA, JA-Ile, SA, H_2_O_2_, or ethylene were responsible for *OsGID1*-mediated rice resistance to BPH, we investigated their levels in oe-GID1 and in WT plants, after infestation by gravid BPH females. Although JA levels accumulated over time following infestation, differences between oe-GID1 and WT plants were slight: Only at 0 h post BPH infestation, JA levels were higher in G11 plants than in WT plants (Figure 5a). Similarly, small differences were observed in JA-Ile: JA-Ile levels in oe-GID1 lines at 24 h after BPH infestation were significantly lower than those in WT plants, whereas, levels of JA-Ile in G1 plants at 0 h and 3 h post BPH infestation were higher than those in WT plants (Figure 5b). The level of H_2_O_2_ in oe-GID1 plants was generally lower than that in WT plants at different time points after infestation (Figure 5c). At 3 h post BPH infestation, SA levels in oe-GID1 plants started to decrease, and this decline continued for 48 h (Figure 5d). Transcript levels of the gene encoding isochorismate synthase (OsICS1), a key enzyme in SA biosynthesis in rice, were consistently lower in plants from G1 and G11 lines, compared to those in WT plants (Figure 5e). In contrast, elicited and constitutive levels of ethylene were higher in plants from G1 and G11 lines than in WT plants (Figure 5f). As oe-GID1 plants were bigger than WT plants (Supplemental Figure S4), levels of ethylene emitted from per g biomass (above-ground parts) of oe-GID1 and WT plants, showed no significant difference (Figure 5f, insert). Consistent with this, relative expression levels of 1-aminocyclopropane-1-carboxylic acid synthase 2 (*OsACS2*), an ethylene biosynthesis-related gene, did not differ over time between oe-GID1 and WT plants, following BPH infestation (Figure 5g).

### 2.6. Overexpression of OsGID1 Decreases the Population Density of Rice Planthoppers and Spiders in the Field

**Figure 6:**
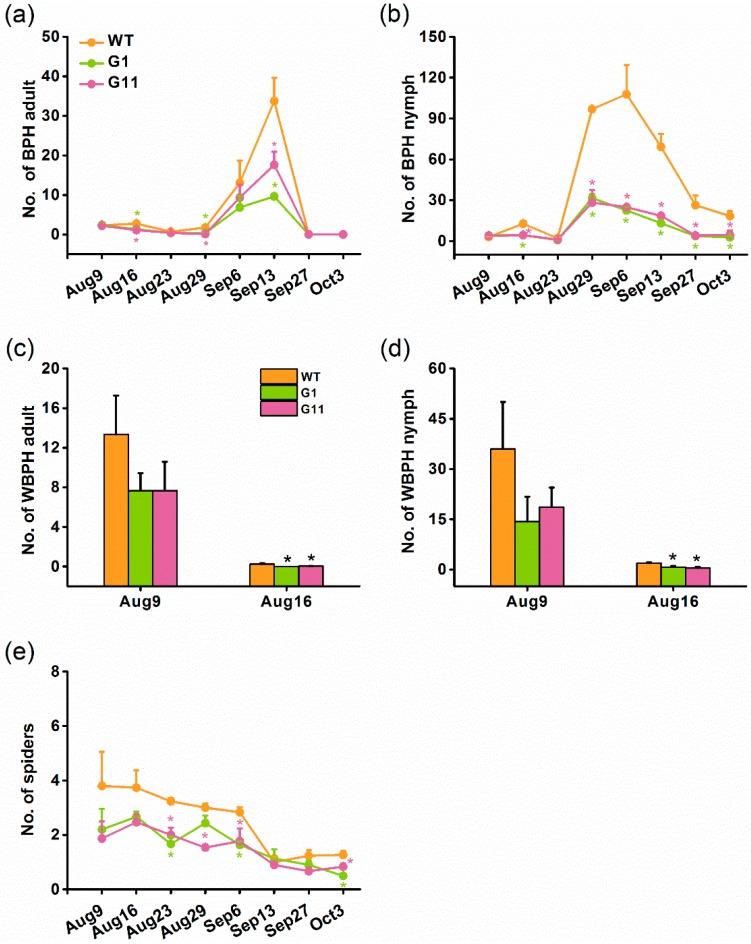
Population dynamics of brown planthopper (BPH), white-backed planthopper (WBPH), and spiders on oe-GID and WT plants in the field. Mean number (+SE, *n* = 3) of BPH adults (**a**), BPH nymphs (**b**), WBPH adults (**c**), WBPH nymphs (**d**), and spiders (**e**) per hill on G1, G11, and WT plants. Asterisks indicate significant differences in oe-GID1 plants, compared with WT plants (Duncan’s multiple range test; * *p* < 0.05).

Since plants with overexpression of *OsGID1* showed enhanced rice resistance to BPH in the laboratory, we questioned whether the overexpression of *OsGID1* also affected the population density of rice planthoppers, and their natural enemies in the field. To answer this question, a field survey of the population dynamics of BPH, white-backed planthopper (WBPH) *Sogatella furcifera*, and predatory spiders was conducted. The population density of BPH adults and nymphs was significantly lower on oe-GID1 plants, compared to those on WT plants (Figure 6a,b). The population density of WBPH adults and nymphs was also lower on oe-GID1 plants than on WT plants; especially on 16 August, the population density of WBPH adults and nymphs on oe-GID1 plants was significantly lower than the population density of WBPH adults and nymphs on WT plants (Figure 6c,d). In the field, the dominant predatory spider species were *Pirata subpiraticus*, *Misumenops tricuspidatus*, *Pardosa pseudoannulata*, and *Tetragnatha maxillosa*. There were fewer spiders on oe-GID1 plants than on WT plants, especially on 23 August, 6 September, and 3 October (Figure 6e).

### 2.7. Overexpression of OsGID1 Reduces Rice Yield in the Field

**Figure 7:**
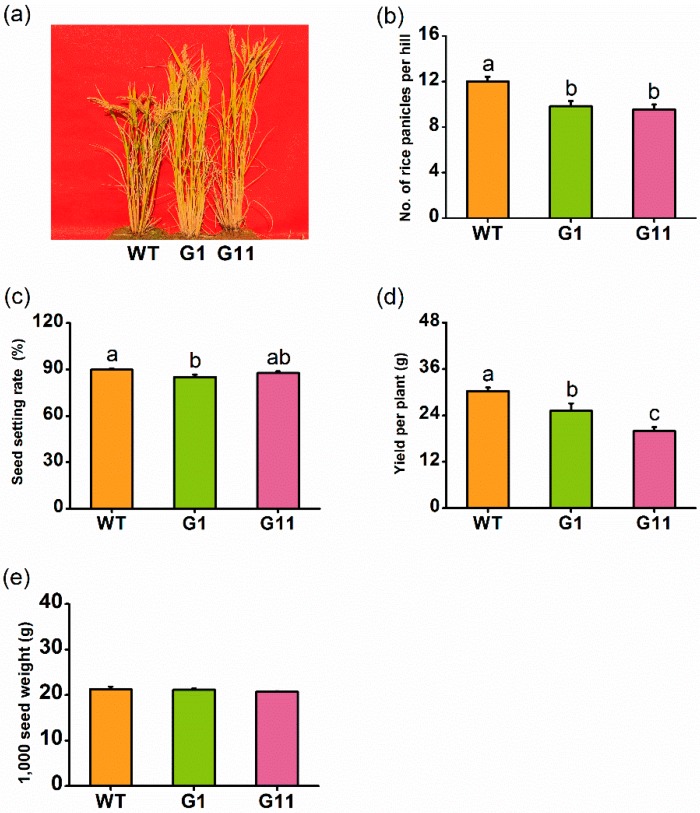
Overexpression of *OsGID1* reduces rice yield in the field. (**a**) Growth genotype of oe-GID1 and WT plants in the field; (**b**) Mean number (+SE, *n* = 30) of panicles per plant of oe-GID1 and WT plants; (**c**) Mean seed setting rate (+SE, *n* = 30) of oe-GID1 and WT plants; (**d**) Mean yield of mature seeds per plant (+SE, *n* = 30) of oe-GID1 and WT plants; (**e**) Mean weight (+SE, *n* = 30) of 1000 seeds of oe-GID1 and WT plants. Different letters indicate significant differences between oe-GID1 lines and WT plants (Duncan’s multiple range test, * *p* < 0.05).

We also investigated the effect of overexpressing OsGID1 on rice yield in the field. Consistent with the growth phenotype reported in Ueguchi-Tanaka et al. [10], oe-GID1 plants showed taller and longer leaves, and fewer tillers in the field, compared with WT plants (Figure 7a). Plants with overexpression of *OsGID1* had far fewer panicles per hill than WT plants (Figure 7b). Although the seed setting rate was similar between WT plants and G11 plants, statistically, it was significantly lower in G1 plants (Figure 7c). The mean weight of mature seeds per WT plant and the 1000-seed weight from WT plants, were clearly heavier than those from oe-GID1 plants (Figure 7d,e). Taken together, these results suggest that the overexpression of *OsGID1* reduces rice yield.

## 3. Discussion

We found that *OsGID1*, in addition to its pivotal role in plant growth and development, also plays an important role in rice defenses. Several lines of evidence support this statement. First, transcript levels of *OsGID1* were induced by BPH infestation, wounding, and SA treatment but not by JA treatment (Figure 1). Second, *OsGID1*-overexpression repressed BPH-elicited levels of three WRKY gene transcripts, SA, and H_2_O_2_, but had a small or no effect on constitutive and elicited levels of ethylene, JA, and JA-Ile (Figure 4 and Figure 5). Since defenses in rice can be induced only by BPH oviposition and not by BPH feeding [38], with no difference found in the number of eggs laid by gravid BPH females on oe-GID1 and WT plants for 12 h (Supplemental Figure S5), the difference in induced defenses stated above between oe-GID1 lines and WT plants should have resulted from the modulation of OsGID1, not from the difference in damage levels. Third, oe-GID1 lines exhibited a GA overdose phenotype and had higher constitutive levels of lignin, a GA responsive marker [24], than WT plants (SupplementalFigure S6), suggesting that the overexpression of *OsGID1* activates the GA-mediated pathway. Fourth, the overexpression of *OsGID1* enhances the resistance of rice to BPH in the laboratory and field (Figure 3 and Figure 6). These findings suggest that *OsGID1*-mediated GA signaling, as we previously reported in References [23,24], positively regulates rice resistance to BPH.

The single GA receptor in rice [10] shows high homology with GID1s from other Poaceae species (Supplemental Figure S2). It has been reported that drought, submergence, and NaCl treatment can rapidly and strongly induce the expression of *OsGID1* [39]. In this study, we observed that infestation by gravid BPH females and wounding, could also induce the expression of *OsGID1*. These data demonstrate that *OsGID1*-mediated GA signaling is influenced by both abiotic and biotic stresses. The trade-off between GA-mediated plant growth and JA- or SA-mediated plant defense has been well documented [7,40,41]. We found that the exogenous application of JA to rice plants had almost no effect on the transcript level of *OsGID1*, and SA treatment had only a weak effect (Figure 1). These findings indicate that the inhibition of plant growth by JA- or SA-mediated plant defenses, may not occur by suppressing transcript levels of the GA receptor gene *OsGID1*.

The GA-signaling pathway is known to influence early events in plant defenses [7,34,42]. For instance, DELLAs can interact directly with the JA ZIM-domain 1 (JAZ1) protein, a key repressor of JA signaling, and EIN3, and thus influence defense responses mediated by JA and ethylene [41,43]. DELLAs can also suppress the accumulation of reactive oxygen species (ROS) and the action of SA, but rather to strengthen JA action in *Arabidopsis* [19,44]. Recently, the DELLA protein in rice OsSLR1 has been observed to enhance transcriptional levels of two herbivore-induced MAPK genes, *OsMPK3* and *OsMPK20-5*, and four WRKY genes, *OsWRKY53*, *OsWRKY70*, *OsWRKY45*, and *OsWRKY24*, as well as the biosynthesis or action of elicited JA, SA, ethylene, and H_2_O_2_ [24,42]. These OsSLR1 actions were thought to occur both directly, by interacting with components of these pathways, and indirectly, through cross-talk among pathways [24]. We did not investigate the effect of the overexpression of *OsGID1* on transcript levels of the MAPK and WRKY genes described above, but we did find that the overexpression of *OsGID1* decreased BPH-induced mRNA levels of three WRKY genes, *OsWRKY13*, *OsWRKY30*, and *OsWRKY33*, as well as induced levels of SA and H_2_O_2_, but increased levels of BPH-elicited ethylene (Figure 4 and Figure 5). Given the close connection between OsGID1, a GA receptor, and OsSLR1, a GA pathway repressor, it is reasonable to think that the same mechanisms may be used to regulate the GA-signaling pathways in plants of both OsGID1 and OsSLR1. Further research should elucidate whether OsGID1 also regulates defense-related MAPK cascades and other WRKYs.

In rice, SA- and H_2_O_2_-mediated signaling pathways positively regulate the resistance of rice to BPH [26,28]; whereas, the ethylene- and JA-mediated signaling pathway negatively regulates rice resistance [27]. However, in this study, we found that the overexpression of *OsGID1* reduced the level of BPH-induced SA and H_2_O_2_ but had a small or no effect on elicited levels of ethylene, JA, and JA-Ile. Thus, the enhanced resistance in oe-GID1 plants to BPH is probably due to factors other than the SA-, H_2_O_2_-, JA- and ethylene-mediated pathways. One of these factors might be lignin, whose levels in plants are positively regulated by the GA pathway [24,45]. Lignin not only strengthens the mechanical hardness of plant tissues and thereby impairs the feeding of herbivores, but it also decreases the digestibility of the food herbivores consume [46]. In rice, it has been reported that silencing *OsSLR1* enhances lignin levels in plants, which then increases the resistance of rice to BPH [24]. Here, we also found that the constitutive level of lignin was higher in oe-GID1 lines than in WT plant (Supplemental Figure S6). The enhanced resistance in plants to BPH may also be due to WRKY TFs, which are modulated by OsGID1, as these have been reported to both positively and negatively regulate plant defenses against herbivores [23,35,47,48]. For example, OsWRKY70 was found to positively modulate the resistance of rice to the striped stem borer (SSB) *Chilo suppressalis* [23], whilst OsWRKY53 negatively modulated rice resistance to SSB [47]. Moreover, silencing *OsWRKY45*, a SA-responsive transcription factor, increased the resistance of rice to BPH, including the decrease in the hatching rate of BPH eggs, suggesting the SA-signaling pathway may negatively affect rice BPH resistance [35]. We observed that BPH-induced mRNA levels of three WRKY genes, *OsWRKY13*, *OsWRKY30*, and *OsWRKY33*, were significantly lower in oe-GID1 plants than in WT plants (Figure 3). These WRKYs are all involved in the activation of the SA-signaling pathway, and in increased plant resistance to pathogens in rice [29,30,31]. *OsWRKY13*, for example, functions as a positive regulator of the SA pathway and a negative regulator of the JA pathway by directly or indirectly modulating the transcription of a subset of genes, which act both upstream and downstream of SA and JA [31]. OsWRKY30, which positively regulates disease resistance in rice, is an SA-inducible gene and can up-regulate *OsWRKY45* [29]. Thus, the decrease in transcript levels of these three WRKY genes may also contribute to the increase in the resistance of oe-GID1 plants to BPH. Further research should investigate the roles of lignin and these WRKYs in the resistance of rice to BPH.

In the laboratory and in the field, oe-GID1 plants showed taller, longer leaves, fewer tillers, and poor yield, compared with WT plants (Figure 7). These observations are consistent with the growth phenotype observed in plants with high levels of GA [10], confirming that the GA-signaling pathway plays a pivotal role in plant growth, development, and reproduction. Interestingly, in the field, we also observed that the population density of spiders on oe-GID1 plants was lower than on WT plants (Figure 7). This difference is probably because most BPH were found on WT plants, and thus a high prey population density will support a high predator population density.

In summary, our data show that the *OsGID*-mediated GA signaling pathway is an important positive regulator of rice resistance to BPH. Our previous results found that infestation by gravid BPH females suppresses the expression of *OsSLR1*, and thus activates the GA-mediated signaling pathway, which in turn induces the production of lignin, and enhances the resistance of rice to BPH [24]. We observed that infestation also activated the GA-signaling pathway by inducing the expression of the GA receptor gene *OsGID1* (Figure 1). Hence, by recognizing signals associated with gravid BPH females, rice can activate the GA pathway in multiple ways, thereby increasing its resistance. Interestingly, compared to mechanical wounding, gravid BPH female infestation induced the expression of OsGID1, weakly and slowly (Figure 1a). This suggested that BPH may suppress the resistance of rice by inhibiting the activation of GA pathway, an interesting question worth studying in the future. Given that the GA-signaling pathway has a negative effect on rice yield, how to balance GA levels so that rice has both a high yield and sufficient resistance to herbivores is also an important topic for future study.

## 4. Materials and Methods

### 4.1. Plant and Insects

The rice genotypes used in this study were Xiushui 110 (XS110; wild type (WT)) (*Oryza sativa* L.) and plants overexpressing *OsGID1*, G1 and G11 (see below). The seedlings of plants from transgenic lines and WT plants were transplanted 10 days after pre-germination to 30 L hydroponic boxes containing a rice nutrient solution [49]. After 4 weeks, seedlings were transferred to 500 mL hydroponic plastic pots, each of which contained one or two plants. Rice plants were used for the experiments 4–5 days after transplanting. The brown planthopper (BPH) *Nilaparvata lugens* colony was originally collected from a rice field in Hangzhou, China, and maintained in growth chambers as previously described in Reference [23].

### 4.2. Structure of OsGID1 and Phylogenetic Analysis

The structure of *OsGID1* was analyzed using a Gene Structure Display Server (GSDS2.0) (http://gsds.cbi.pku.edu.cn). The SNPs in the CDS of *OsGID1* were analyzed after submitting its accession number to the National Center of Biotechnology Information (NCBI, https://www.ncbi.nlm.nih.gov/SNP/). The protein sequences of OsGID1, and its homologs from different plant species were downloaded from the NCBI (http://www.ncbi.nlm.nih.gov). Sequences were then aligned by ClustalW method, using the default parameters in MEGA X, as described in Reference [50]. The neighbor-joining method was used to construct the phylogenetic tree using bootstrap analysis with 1000 replicates through MEGA X [51].

### 4.3. Generating Transgenic Plants

The full-length coding sequence of *OsGID1* (TIGR ID Os05g33730) was PCR-amplified using a pair of primers, GID1-F (5′-GTCGACAATCTCCTCCCTTCTCGA-3′) and GID1-R (5′-ACTAGTCTAGTAGTAGAGGTTA-3′), from a cDNA library of rice variety XS110. The sequence-confirmed products were cloned into the binary vector pCAMBIA1301, yielding an overexpression transformation vector pCAMBIA1301-*OsGID1*. The expressing vector was introduced into rice variety XS110 via *Agrobacterium tumefaciens*-mediated transformation. Screening of homozygous T2 plants and Southern blotting were performed as described in Reference [28]. Two homozygous *GID1*-overexpressing lines of the T2 generation with a single insertion (G1 and G11) were used for further experiments.

### 4.4. RNA Extraction and Quantitative Real-time PCR

For gene expression analysis, five independent biological samples were used. Total RNA was extracted from 100 mg of rice stem tissues using SV Total RNA Isolation System (Promega, Madison, WI, USA), and RNA was reverse-transcribed using a PrimeScript^™^ RT reagent Kit (Perfect Real Time) (TaKaRa, Dalian, China), following the manufacturer’s protocols. Quantitative real-time PCR (qRT-PCR) was carried out using a CFX96TM Real-Time System (Bio-Rad, Hercules, CA, USA) with Premix Ex Taq^™^ Kit (TaKaRa, Dalian, China). A linear standard curve, threshold cycle number versus log (designated transcript level), was built using a series concentrations of a specific cDNA standard. Relative levels of the transcript of the target gene in tested samples were calculated according to the standard curve. A rice actin gene *OsACT* (TIGR ID: Os03g50885) was used as an internal standard to normalize cDNA concentrations. Specific primers and probe sequences used for qRT-PCR are provided in Table S1.

### 4.5. Plant Treatments

For mechanical wounding treatments, the lower sections of rice stems were pierced 200 times with a fine needle. Control plants were left untreated. For BPH treatment, individual plants were infested with 15 gravid BPH females, enclosed in a glass cylinder (4 × 8 cm, with 48 small holes, 0.8 mm in diameter). An identical glass cylinder without BPH was enclosed for control plants (non-infested). For JA and SA treatments, rice plants were sprayed individually with 2 mL of JA (100 µg mL^−1^) or SA (70 µg mL^−1^) in 50 mM sodium phosphate buffer. Rice plants sprayed with 2 mL of the buffer were used as controls. Five replications for each treatment, at each time point, were carried out.

### 4.6. JA, JA-Ile, SA and Ethylene Measurements

Under JA, JA-Ile, and SA analysis, oe-GID1 and WT plants were randomly assigned to BPH and control (non-infestation) treatments. The leaf sheaths of each plant were harvested at 0 h, 3 h, 8 h, 24 h, and 48 h, post BPH infestation. Samples were mixed with ethyl acetate containing labelled internal standards, and then analyzed using a HPLC/mass spectrometry/mass spectrometry system to quantify JA, JA-Ile, and SA levels in each plant as previously described in Reference [24]. For ethylene analysis, plants from each line were individually covered with a sealed glass cylinder (4 cm in diameter and 50 cm in height), into which 15 gravid BPH females were released. Ethylene production was measured at 3 h, 6 h, 12 h, 24 h, 48 h, and 72 h post BPH infestation, following the same method as described in Lu et al. [36]. Experiments for each treatment, at each time point, were replicated five times.

### 4.7. Hydrogen Peroxide and Lignin Content Analysis

WT and oe-GID1 plants were randomly assigned to treatment with BPH infestation or non-infestation. The leaf sheaths of each plant were harvested at 0 h, 3 h, 8 h, and 24 h, post BPH infestation. Samples were stored at −80 °C until use. Hydrogen peroxide concentrations were measured using a Amplex^®^ Red Hydrogen Peroxide/Peroxidase Assay Kit (Invitrogen, Eugene, OR, USA), following the manufacturer’s protocol. The leaf sheaths of 4-week-old rice plants from oe-GID1 and WT plants were harvested, and lignin contents were determined, as described in Xu et al. [51]. Experiments for each treatment, at each time interval, were replicated five times.

### 4.8. BPH Performance Assay

To investigate the effect of oe-GID1 plants on the feeding and oviposition preference of gravid BPH females, stems of two plants, either a G1 or G11 plant and a WT plant, were covered with a glass cylinder (4 × 8 cm, with 48 small holes, 0.8 mm in diameter), into which 15 gravid BPH female adults were introduced. The number of BPH adults on each plant was counted at 1, 2, 4, 8, 24, and 48 h, after the insects had been released. Adults were removed 48 h after infestation, and the eggs laid on each plant were examined with a microscope. The experiment was replicated ten times.

To determine the influence of oe-GID1 plants on the hatching rate of BPH eggs, 15 gravid BPH females were allowed to oviposit on individual plants of oe-GID1 and WT lines for 12 h. Every day newly hatched BPH nymphs on each plant were recorded, until no new nymphs were observed for three consecutive days. Unhatched eggs were examined with a microscope. Based on these data, the developmental duration and hatching rate of BPH eggs were calculated. Each line was replicated ten times.

### 4.9. Field Experiment

To assess the resistance of *OsGID1*-overexpressing plants and their corresponding WT plants to insects in a natural environment, a field experiment was performed in Changxing, Zhejiang, China. The field plot consisted of 9 blocks (6.5 m in length and 3.5 m in width), each of which was surrounded by a 0.5 m buffer zone. On the plot, plants from three lines were randomly assigned to 9 blocks; each line with three independent replicate blocks. The number of adults and nymphs of BPH and WBPH, as well as the number of spiders, the main predators of herbivores, were recorded once a week from June to October in 2014. To collect these data, we randomly sampled 10 hills of plants, in each plot, at each time interval.

To evaluate the effect of overexpressing *OsGID1* on rice yield in the field, 10 hills of mature plants per block were randomly harvested. After being sun dried, the productivity-related parameters—the number of rice panicles per hill, seed setting rate, yield per plant, and 1000-seed weight—were recorded in the laboratory. The seed setting rate was assessed as the ratio of mature seeds to the total number of grains (including abortive grains); yield per plant equaled to the total weight of mature seeds per plant; 1000-seed weight was calculated as follows: 1000-seed weight = total weight of mature seeds/total number of mature seeds × 1000.

### 4.10. Statistical Analysis

Differences in the relative expression levels of *OsGID1* in response to different treatments were determined using the Student’s *t*-test. Differences in the feeding and oviposition preference of gravid BPH females were determined by the chi-square test. Differences in other data were analyzed by one-way ANOVA; if the ANOVA analysis was significant (*p* < 0.05), Duncan’s multiple range test was used to detect significant differences among groups. When necessary, data were log-transformed or arcsine-transformed to meet requirements for the homogeneity of variance. All tests were carried out with Statistica (SAS Institute, Inc., Cary, NC, USA).

## Supplementary Materials

Supplementary materials can be found at http://www.mdpi.com/1422-0067/19/9/2744/s1. Supplemental Figure S1: The structure of *OsGID1* and single nucleotide polymorphisms in its coding sequence; Supplemental Figure S2: Phylogenetic analysis of GID1 proteins from different plant species; Supplemental Figure S3: DNA gel-blot analysis of oe-GID1 lines and WT plants; Supplemental Figure S4: Mean mass (+SE, *n* = 20) of the above-ground part of 38-day-old plants of oe-GID1 and WT lines; Supplemental Figure S5: Mean number (+SE, *n* = 5) of eggs laid by 15 gravid BPH females on oe-GID1 and WT plants for 12 h. Supplemental Figure S6: Constitutive levels of Lignin in WT and oe-GID1 plants; Table S1: Primers and probes used for qRT-PCR of target genes.
