# Supplementary material for: Overexpression of OsGID1 Enhances the Resistance of Rice to the Brown Planthopper Nilaparvata lugens
Source: Int J Mol Sci. 2018 Sep 13;19(9):2744. doi: 10.3390/ijms19092744 (PMC6164479; doi:10.3390/ijms19092744)
Supplement: Supplementary file 1 [file ijms-19-02744-s001.pdf]

Supplementary Materials: **Overexpression of *OsGID1* Enhances the Resistance of Rice to the Brown Planthopper *Nilaparvata lugens***

Lin Chen, Tiantian Cao, Jin Zhang and Yonggen Lou

State Key Laboratory of Rice Biology & Ministry of Agriculture Key Lab of Molecular Biology of Crop Pathogens and Insects, Institute of Insect Sciences, Zhejiang University, Hangzhou 310058, China

Correspondence: Prof. Yonggen LOU, yglou@zju.edu.cn

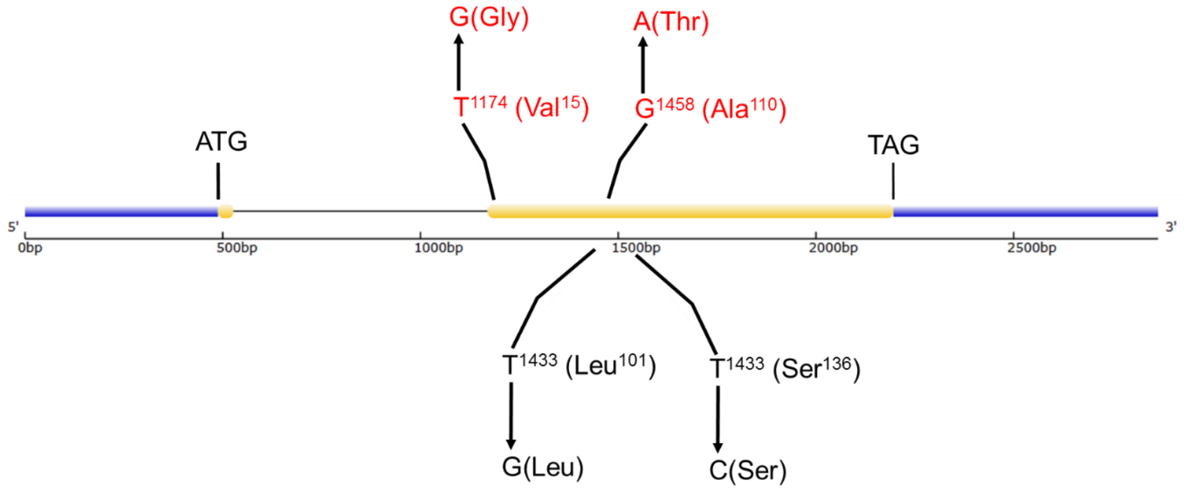

Supplemental Figure S1. The structure of *OsGID1* and single nucleotide polymorphisms (SNPs) in its coding sequence. The *OsGID1* consists of two exons (yellow boxes) and one intron (thin line); upstream and downstream regions, including untranslated regions, are showing in blue boxes. Missense and synonymous nucleotide substitutions of the four SNPs are indicated in red and dark.

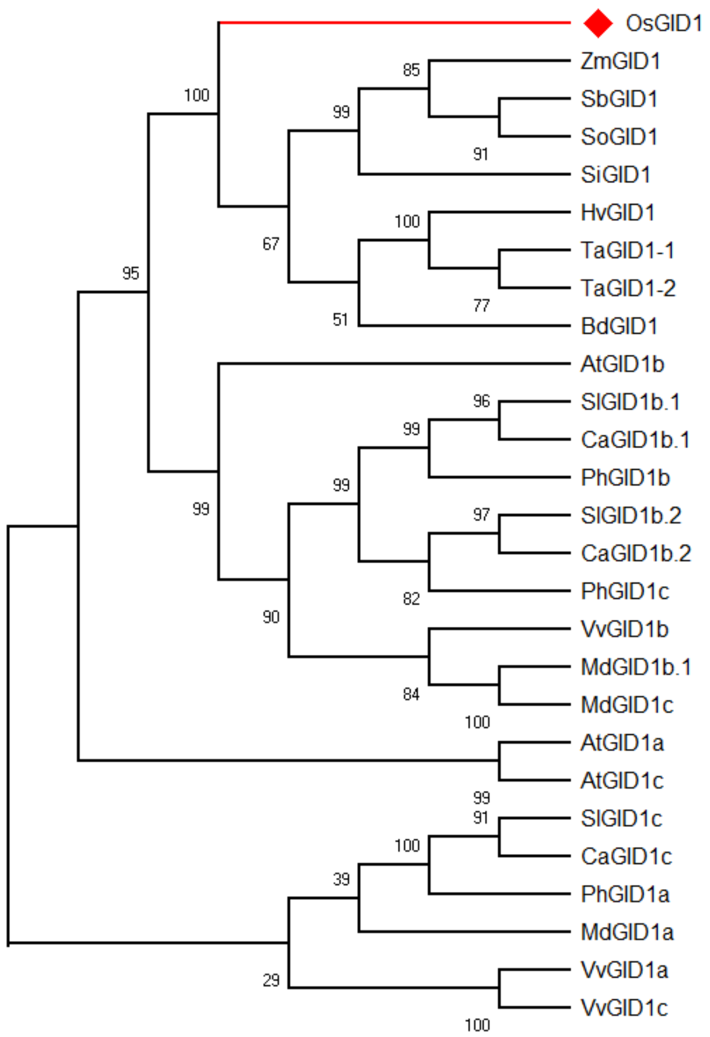

Supplemental Figure S2. Phylogenetic analysis of GID1 proteins from different plant species. The unrooted tree was constructed with a neighbor-joining method on the basis of the alignment of protein sequences and confirmation of the tree topology by bootstrap analysis (1,000 replicates) were performed with MEGA X software (default settings except the replicates of the bootstrap value). Species acronyms are included before the protein name: Os, *Oryza sativa*; At, *Arabidopsis thaliana*; Zm, *Zea mays*; Hv, *Hordeum vulgare*; Si, *Setaria italic*; Sb, *Sorghum bicolor*; So, *Saccharum officinarum*; Bd, *Brachypodium distachyon*; Ta, *Triticum aestivum*; Sl, *Solanum lycopersicum*; Vv, *Vitis vinifera*; Md, *Malus domestica*; Ca, *Capsicum annuum*; Ph, *Petunia x hybrid*. Sequence data in the phylogenic tree can be found in the GenBank/EMBL data libraries under accession numbers: OsGID1 (NP_001055520.1); AtGID1a (NP_187163.1), AtGID1b NP_191860.1, AtGID1c (NP_198084.1); ZmGID1 (CAP64327.1); HvGID1 (CAO98733.1); SiGID1 (XP_004962116.1); SbGID1 (XP_021303311.1); SoGID1 (CAP64326.1); BdGID1 (XP_003568469.1); TaGID1-1 (CBW30246.1), TaGID1-2 (CBW30247.1); SlGID1b.1 (XP_004240525.1), SlGID1b.2 (NP_001234767.2), SlGID1c (XP_004230154.1); VvGID1a (AFG17072.1), VvGID1b (XP_002271700.1), VvGID1c (XP_002265764.1); MdGID1a (NP_001281280.1), MdGID1b (AFD32891.1), MdGID1c (AFD32892.1); CaGID1b.1 (ALY05368.1), CaGID1b.2 (ALY05369.1), CaGID1c (ALY05370.1); PhGID1a (AGN72648.1), PhGID1b (AGN72649.1), PhGID1c (AGN72650.1). The OsGID1s is indicated with red ◆.

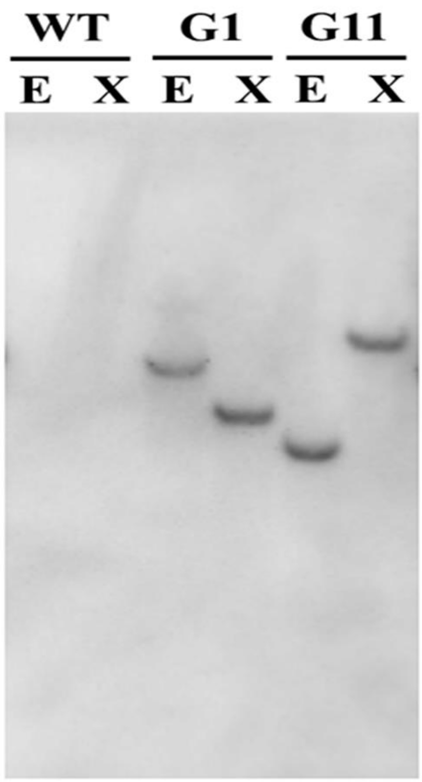

Supplemental Figure S3. DNA gel-blot analysis of oe-GID1 lines, G1, G11 and WT plants. Genomic DNA extracted from oe-GID1 and WT plants was digested with *Eco*R I (E) and *Xba* I (X). The blot was hybridized with a probe specific for reporter gene *GUS*. All overexpression lines have a single insertion of *OsGID1*.

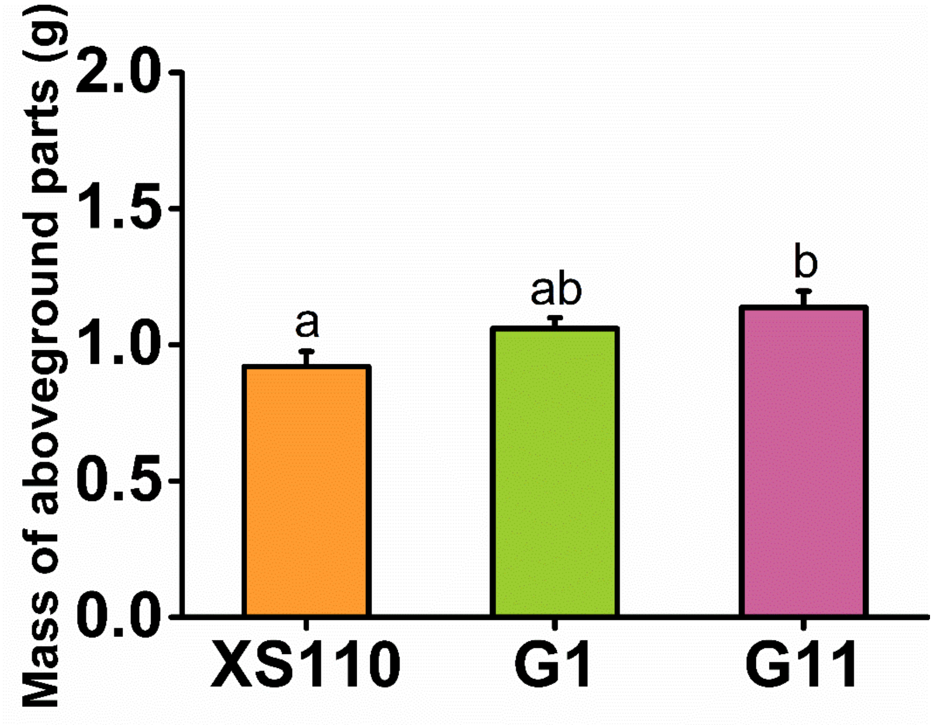

Supplemental Figure S4. Mean mass (+ SE, n = 20) of the above-ground part of 38-day-old plants of oe-GID1 and WT lines. Different letters indicate significant differences among lines (Duncan's multiple range test, $P < 0.05$).

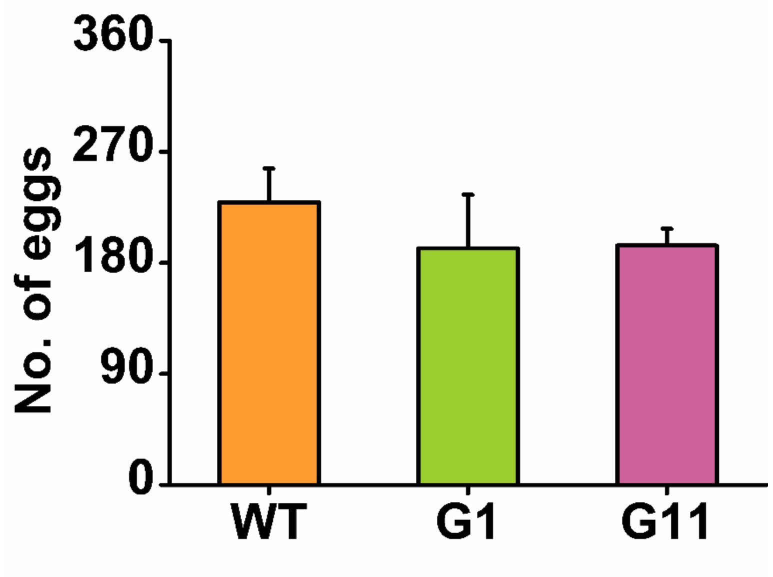

S**upplemental Figure S5.** Mean number (+ SE, n = 5) of eggs laid by 15 gravid BPH females on oe-GID1 and WT plants for 12 h.

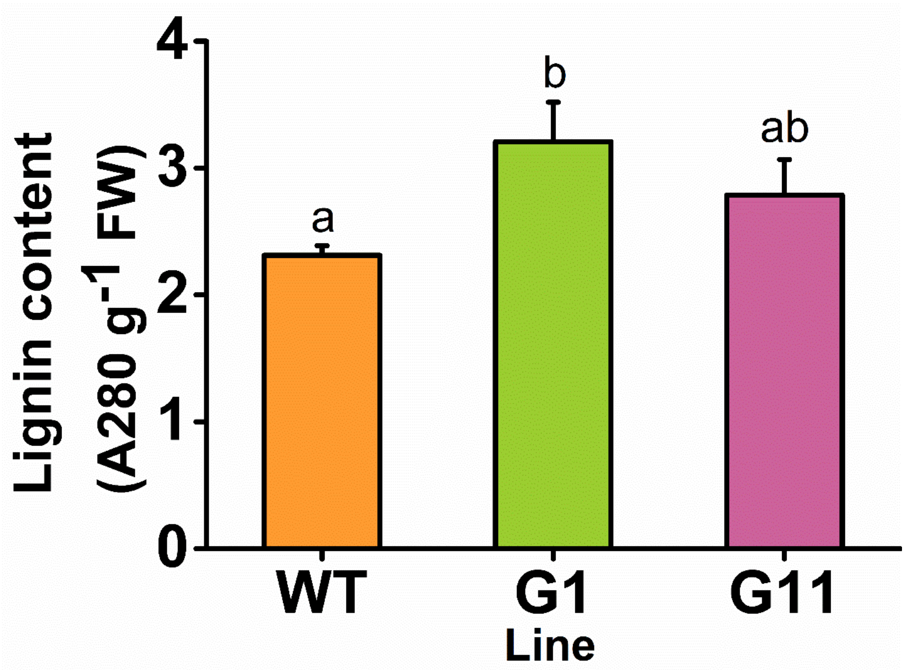

Supplemental Figure S6. Constitutive levels of Lignin in WT and oe-GID1 plants.

Mean constitutive levels (+SE, n = 5) of lignin in leaf sheaths of 4-week-old plants of oe-GID1 and WT lines. Different letters indicate significant differences among lines (Duncan's multiple range test, $P < 0.05$).

Table S1. Primers and probes used for qRT-PCR of target genes

Gene	TIGR ID	Primer (5′-3′)	Probe (5′-3′)
ACTIN	Os03g50885	FP: TGGACAGGTTATCACCATTGGT	CGTTTCCGCTGCCCTGAGGTCC-BHQ1
		RP: CCGCAGCTTCCATTCCTATG	
OsWRKY13	Os01g54600	FP: GCGCAAGTACGGCCAGAA	CCCATCAAGGGCTCTCCCTACCCA
		RP: CCTTGGAGCTACTGCACCTGTA	
OsWRKY30	Os08g38990	FP: AACAGTGGCCACCCAAGCT	AGGCTCAGCCGCTGCACCACA
		RP: GTTCAGGTCTCCGGTGAAGAAG	
OsWRKY33	Os03g33012	FP: AGGCAAGCACAGCCATGAC	CCAGCAGCCAGGAACAGTAGCCATTC
		RP: GAAGACGATACGTTGGCATTAGC	
OsGID1	Os05g33730	FP: ACCGCAACGAGTGCAAGAC	CCGCTCCACACATGGGTGCTCA
		RP: TTGTACGACAGCTTGAAGTTGGA	
OsICS1	Os09g19734	FP: ACCAATTATGTTTCCGATCAATCA	ACACCTCCTACATACGCAGCCGCTGA
		RP: CGTCGCCTTCTTGGATTTATG	
OsC4H	Os01g60450	FP: GTGTACCGCATCATGTTC	
		RP: GAAGTCGCCGTAGTTGTA	
OsACS2	Os04g48850	FP: CACCCCGAGGCATCCAT	TGCACACCGGAGGGCGTCT
		RP: ATTGGCGATCCTCTTGAACTG	
